# Gelsolin suppresses tumorigenicity through inhibiting PKC activation in a human lung cancer cell line, PC10

**DOI:** 10.1038/sj.bjc.6600739

**Published:** 2003-02-18

**Authors:** N Sagawa, H Fujita, Y Banno, Y Nozawa, H Katoh, N Kuzumaki

**Affiliations:** 1 Division of Cancer Gene Regulation, Research Section of Disease Control, Institute for Genetic Medicine, Hokkaido University, N15 W7 Kita-ku, Sapporo 060-0815, Japan; 2 Surgical Oncology, Cancer Medicine, Division of Cancer Medicine, Hokkaido University Graduate School of Medicine, Sapporo 060-8638, Japan; 3 Department of Biochemistry, Gifu University School of Medicine, Tsukasamachi-40, Gifu 500-8706, Japan; 4 Department of Environmental Cell Responses, Gifu International Institute of Biotechnology and Institute of Applied Biochemistry, Mitake, Gifu 505-0116, Japan

**Keywords:** gelsolin, phosphoinositides, PKC, lung cancer, tumour suppression

## Abstract

Gelsolin expression is frequently downregulated in lung cancer and several types of different human cancers. To examine the effects of gelsolin restoration on tumorigenicity, we here stably expressed various levels of gelsolin via gene transfer in lung cancer cells (squamous cell carcinoma line, PC10). We observed the alterations in tumorigenicity *in vivo* when implanted in nude mice, and the changes in growth properties *in vitro*. As compared to parental cells and control clones, gelsolin transfectants highly reduced tumorigenicity and repressed cell proliferation. Moreover, we investigated bradykinin-induced responses in gelsolin-overexpressing clones, because agonist-stimulated activation of the phospholipases C (PLC)/protein kinase C (PKC) signal transduction pathway is critical for cell growth and tumorigenicity. Bradykinin promotes phosphatidylinositol 4,5-bisphosphate (PIP2) hydrolysis by PLC and translocation of various PKC isoforms from the cytosolic fraction to the particulate fraction. Bradykinin treatment did not increase inositoltriphosphate (IP3) production and induce the membrane fractions of PKC*α* and PKC*γ* in gelsolin tranfectants, while it induced PIP2 hydrolysis and increased the fractions in parental and control clones. These results suggest that gelsolin suppressed the activation of PKCs involved in phospholipid signalling pathways, inhibiting cell proliferation and tumorigenicity.

The long-term accumulation of genetic aberrations plays a major role in the development of various cancers. Molecular abnormalities include expression of oncogenes, such as Ki-ras, c-*myc*, c-*erb*-B-2 and *bcl*-2, and tumour suppressor genes, such as *p53*, *Rb* and *FHIT* (fragile histidine triad) in lung cancer (Otterson *et al*, 1998; Salgia and Skarin, 1998).

We previously demonstrated that expression of an actin-regulatory protein (Yin and Stossel, 1979), gelsolin, is frequently downregulated in lung cancer (Dosaka-Akita *et al*, 1998) and several types of different human cancers, such as stomach, bladder and colon (Moriya *et al*, 1994; Tanaka *et al*, 1995; Furuuchi *et al*, 1996). Moreover, we indicated that introducing human cytoplasmic gelsolin cDNA suppresses tumorigenicity in human bladder and colon cancer cell lines (Tanaka *et al*, 1995; Furuuchi *et al*, 1996). Gelsolin controls the length of actin polymers *in vitro* by a variety of mechanisms. At least three different activities are known: severing, capping and nucleating through interaction with both filamentous (F-) and monomeric (G-) actins are responsible for reorganisation of the actin cytoskeleton. These functions are tightly regulated by calcium ions (Ca^2+^), pH and polyphosphoinositides (Janmey, 1994). Although gelsolin may modulate phospholipid signaling pathways through its high affinity to polyphosphoinositides (Janmey and Stossel, 1987), the mechanism that restored gelsolin expression suppresses tumorigenicity in cancer cells is not well understood.

Protein kinase C (PKC) belongs to a ubiquitous family of serine/threonine kinases that plays a critical role in many signal transduction pathways (Nishizuka, 1984). PKCs are activated by a variety of extracellular stimuli that elicit production of a lipid second messenger diacylglycerol (DAG) and a cofactor phosphatidylserine (PS) (Kishimoto *et al*, 1980; Nishizuka, 1992; Toker, 1998). Up to 12 different PKC isoforms have been reported in mammalian cells (Dekker and Parker, 1994; Mellor and Parker, 1998). The isoforms have been divided into three distinct subfamilies: the conventional PKCs (cPKC*α*, *β*I, II and *γ*), which are activated by DAG, PS and Ca^2+^; novel PKCs (nPKC*δ*, *ɛ, η, θ* and *μ*), which are dependent on DAG and PS, but not on Ca^2+^; and atypical PKCs (aPKC*ζ*, *ι/λ*), which are activated by PS, but not by DAG and Ca^2+^ (Toker *et al*, 1994). Several studies have indicated that the individual PKC isoforms express in their tissue specifically and vary in biochemical properties and intracellular localisation (Nishizuka, 1992).

PKCs are shown to be a target for phorbol ester, such as 12-*O*-tetradecanoylphorbol-13-acetete (TPA) which is known as a tumour promoter, because TPA contains a diacylglycerol-like structure (Castagna *et al*, 1982; Gopalakrishna and Barsky, 1988). Several studies showed that overexpression of different PKC isoforms induces high growth rates, cellular saturation densities and enhanced tumorigenicity (Housey *et al*, 1988; Persons *et al*, 1988; Cacace *et al*, 1993; Mischak *et al*, 1993; Borner *et al*, 1995). Furthermore, the transfection of antisense PKC*α* into lung cancer cells repressed cell proliferation and reduced tumorigenicity in nude mice (Wang *et al*, 1999). However, there are a number of conflicting reports that overexpression of PKC increases or suppresses tumorigenicity in various cells (Janik *et al*, 1994; Johnson *et al*, 1999). These observations suggest that various PKCs play various roles in carcinomas and distinctive roles in cell regulation.

In this study, we established lung cancer cells (squamous cell carcinoma, PC10) overexpressing human cytoplasmic gelsolin in various degrees by gene transfer, using retrovirus carrying human cytoplasmic gelsolin cDNA, and examined the effects of restoration of gelsolin expression on tumorigenicity *in vivo* with nude mice and growth properties in culture *in vitro*. Moreover, we investigated bradykinin-induced responses, particularly inositoltriphosphate (IP3) production and translocation of various PKC isoforms from the cytosolic fraction to the particulate fraction in gelsolin-overexpressing clones, because agonist-stimulated activation of the phospholipases C (PLC)/PKC signal transduction pathway is critical for cell proliferation and tumorigenicity.

## MATERIALS AND METHODS

### Materials

Geneticin (G418) and LipofectAMINE were purchased from Life Technologies. Bradykinin and anti-human gelsolin monoclonal antibody (clone: GS-2C4) were from Sigma. An anti-actin monoclonal antibody was from Boehringer Mannheim Biochemica. Monoclonal antibodies to PKC isoforms were from Transduction Laboratories. Chemiluminescence kit (ECL system) was from Amersham Pharmacia Biotech.

### Cell culture

Human lung cancer cell PC10 (squamous cell carcinoma) obtained from the Health Science Research Resources Bank of Japan (Osaka, Japan) were grown in RPMI 1640, supplemented with 10% foetal calf serum (FCS; Life Technologies) and 0.03% L-glutamine. Culture was maintained at 37°C in a moist atmosphere of 95% air and 5% CO_2_.

### Retrovirus construction for human cytoplasmic gelsolin and gene transfer

Retroviral vector for the expression of human cytoplasmic gelsolin (pLNChGsn) was constructed as described previously with pLNCX (a generous gift from Dr AD Miller, Fred Hutchinson Cancer Research Center) (Banno *et al*, 1999). Amphotropic retrovirus containing gelsolin cDNA and empty virus were obtained by transfection of each vector into CAK8 (kindly provided by Dr Karl Riabowol, University of Calgary), a highly efficient virus-packaging cell line, using LipofectAMINE. At 2 days after transfection, virus-containing supernatants were recovered, spun with 1200 r.p.m. and then used for infection with PC10 cells. Infected cells were cultured in a selection medium containing 400 *μ*g ml^−1^ G418 for 14–20 days and individual colonies were picked randomly and then expanded.

### Western blotting and densitometric quantification

Total cell lysates were prepared by extracting the cell pellets with RIPA buffer (50 mM HEPES, 50 mM NaCl, 0.1% SDS, 1% NP-40, 2 mM EDTA, 2 mM EGTA, 1 mM phenylmethylsulphonyl fluoride (PMSF), 5 *μ*g ml^−1^ leupeptin and 10 *μ*g ml^−1^ aprotinin). The protein concentration was determined by the Bio-Rad protein assay kit (Bio-Rad, Richmond, CA, USA) with bovine serum albumin (BSA) as a standard. A portion equivalent to 20 *μ*g of protein was separated by SDS–PAGE and electrotransferred to nitrocellulose membrane. After blocking with 5% nonfat dry milk in TBST (Tris-buffered saline: 10 mM Tris-HCl, pH 8.0 and 150 mM NaCl containing 0.05% Tween-20), they were probed with anti-human gelsolin antibody (GS-2C4), 2nd antibody and then visualised with the ECL system. Band images were scanned with a GT-6000 Scanner (Epson-SEIKO) and densitometric analyses were performed using NIH Image, ver. 1.56. Proteins were normalised against the levels of actin protein.

### Growth assay in monolayer culture and MTT assay

Cells were seeded at a density of 5×10^4^ cells per well in six-well plates in RPMI 1640 with either 10 or 1% FCS, respectively. This point was designated day 0. Cells number in triplicate wells was determined by counting with a haemocytometer after trypsinisation every 48 h. Flat 96-well culture plates seeded at a density of 5×10^3^ cells per well were used to test growth with 1% FCS medium by MTT assay. MTT was dissolved at 5 mg ml^−1^ in phosphate-buffered saline solution and used. After 40 h, 10 *μ*l of MTT solution was directly added to the medium and the cells were incubated for an additional 6 h. After removal of the medium, 100 *μ*l of 0.04 N hydrochloric acid in isopropanol was added to each well and the optical density of the plates was measured on a microculture plate reader (Spectra Max 250; Molecular Devices) using a test wavelength of 570 nm and a reference wavelength of 630 nm.

### Cell death assay

Cells were treated with staurosporine (STS; Sigma), a potential inducer of apoptotic cell death, at 250 ng ml^−1^ and incubated for various intervals. Cell death was examined with fluorescence microscopy (TE-300; Nikon) for nuclear morphological changes such as a nuclear condensation after an addition of Hoechst 33342 at a final concentration of 1 *μ*g ml^−1^. Apoptotic index was determined by counting the number of apoptotic cells with a nuclear condensation divided by the total number of cells (at least 300 cells) in the fields.

### Anchorage-independent growth assay

For the assay of colony-forming efficiency in soft agar, 5×10^3^ cells in 1 ml growth medium containing 0.33% Noble agar (DIFCO Laboratories, Detroit, MI, USA) were incubated in a 60 mm dish with glids (Coster Scientific Co. Corning, NY, USA) overlaid on 4 ml of 0.5% agar medium. Cells were incubated at 37°C in a moist atmosphere of 95% air and 5% CO_2_ and 3 weeks later the number of colonies was counted using an inverted phase microscope. The data are expressed as colony-forming efficiency =[(number of colonies ×100)/(number of cells originally seeded)].

### Tumorigenicity in nude mice

Cells were trypsinised from monolayer cultures, counted, and spun down with 1500 r.p.m. for 5 min and resuspended with RPMI 1640. Two BALB/c-nu/nu syngenic 6-week-old male mice (Japan SLC, Inc. Shiruoka, Japan) per clone were injected subcutaneously into the back with 3×10^6^ cells of gelsolin transfectants, neo-control or parental PC10 cells in a final volume of 100 *μ*l of PBS. Tumour size was measured using a hand calipers every 10 days. Volumes were calculated by the formula 1/2×*L*×*W*^2^ where *L* and *W* are length and width, respectively, of the tumour measured in two dimensions. The animal experiments were conducted under the guidelines for the use of experimental animals laid down by the Hokkaido University School of Medicine and the United Kingdom Co-ordinating Committee on Cancer Research (UKCCCR) guidelines for the welfare of animals in experimental neoplasia (Workman *et al*, 1998).

### Assay for phospholipase C (PLC) activity

PLC activity was assessed by the measurement of total inositol phosphates. Cells in six-well plates were prelabelled with 1 *μ*Ci ml^−1^ myo-[^3^H] inositol for 24 h in inositol-free RPMI 1640 medium containing 0.3% BSA. Cells were then washed twice with the HEPES/Tyrode buffer (10 mM HEPES/NaOH, pH 7.4, 134 mM NaCl, 12 mM NaHCO_3_, 2.9 mM KCl, 0.36 mM NaH_2_PO_4_, 1.0mM MgCl_2_, 1.8 mM CaCl_2_ 1 mg ml^−1^ BSA and 1 mg ml^−1^ glucose) containing 20 mM LiCl and further incubated for 15 min at 37°C before stimulation with agonists. Cells were then stimulated with bradykinin at the indicated concentration for 2 min, and the reactions were terminated by the addition of ice-cold 10% perchloric acid. Inositol phosphates were separated using AG 1×8 anion exchange resin (formate form, 200–400 mesh, Bio-Rad) as described previously (Berridge *et al*, 1983).

### Protein kinase C (PKC) translocation assay

Gelsolin transfectants, neo-control and parental PC10 cells were cultured to 70–80% confluence on 150 mm plates in complete medium (RPMI 1640, supplemented with 10% FCS and 0.03% L-glutamine). The cells were starved by serum-free RPMI 1640 medium for 48 h before stimulation. After starvation, the cells were exposed to bradykinin (100 nM) for 5 min at 37°C in a moist atmosphere of 95% air and 5% CO_2_. The cells were washed with ice-cold phosphate-buffered saline, harvested with scraper, and cell pellets were lysed with homogenisation buffer (20 mM Tris-HCl, pH 7.4, 250 mM sucrose, 2 mM EDTA, 10 mM 2-mercaptoethanol, 1 mM PMSF, 5 *μ*g ml^−1^ leupeptin and 10 *μ*g ml^−1^ aprotinin) by Dounce homogenisation (50 strokes). The homogenate was centrifuged at 200 ***g*** for 10 min to removed nuclei and unlysed cell, and the resulting supernatant was centrifuged at 100 000×***g*** for 1 h. The supernatants (soluble fraction) were analysed for protein content and prepared for electrophoresis (Rotenburg and Sun *et al*, 1998). The pellets (particulate fraction) were resuspended with RIPA buffer containing 0.1% Triton X-100, placed at 4°C overnight. Soluble (cytosol) and particulate (membrane) fractions (20 *μ*g per lane) were separated with 10% of polyacrylamide gel and analysed by Western blot analysis with monoclonal antibodies specific for PKC isoforms.

## RESULTS

### Establishment of lung cancer cell line overexpressing gelsolin

**Figure 1:**
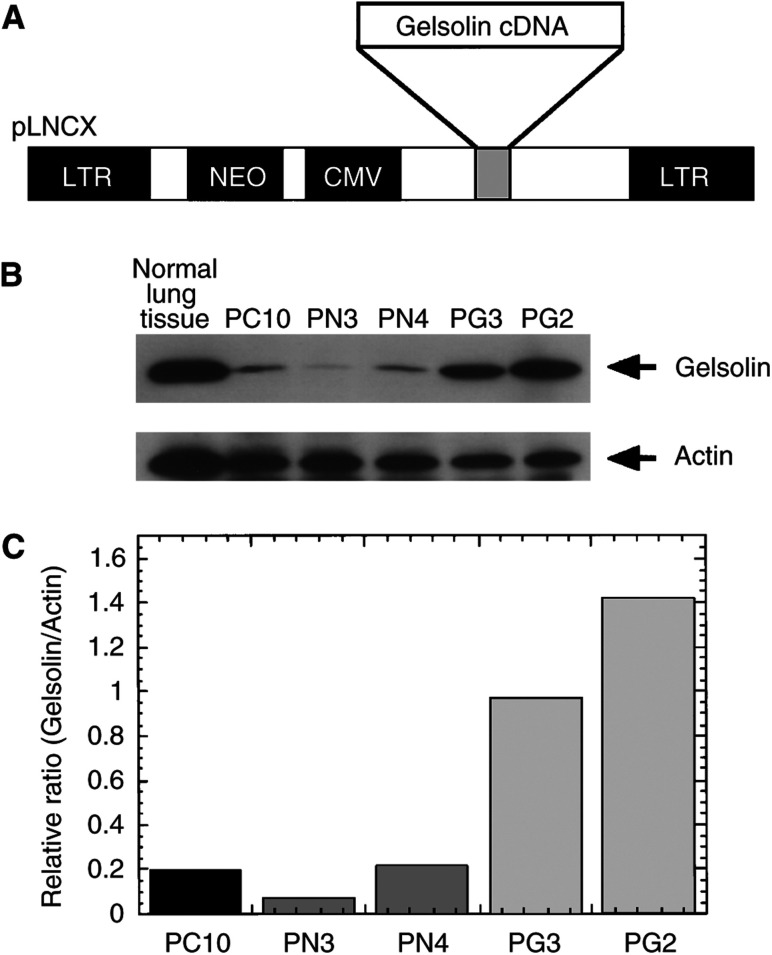
Identification of human lung cancer cells transfected with gelsolin. (**A**) Construction of gelsolin-overexpressive clones. The human cytoplasmic gelsolin cDNA was cloned into the *Hind*III–*Stu*I site of pLNCX retoroviral vector as described in Materials and Methods. The resulting construct, pLNChGsn or pLNCX alone was transfected into CAK8, a highly efficient ecotropic virus-packaging cell line. (**B**) Western blot analysis of the gelsolin expression in stably transfected clones. PC10 cells were transfected with pLNCX or pLNChGsn, and stable transfectants were established after selection with G418 as described in Materials and Methods. Parental (lanes 1), neo-transfectants (lanes 2 and 3) and gelsolin transfectants (lanes 4 and 5) are shown. (**C**) Densitometric quantification of protein levels in transfectants is shown after normalisation against the levels of actin protein using NIH Image.

The protein level of gelsolin in PC10 cells was 1/30th in comparison with that of normal lung tissues on Western blot analysis (Dosaka-Akita *et al*, 1998). To stably transduce gelsolin into lung cancer cells, we obtained amphotropic retrovirus carrying the human cytoplasmic gelsolin cDNA (designated as RGV) through transient transfection of CAK8 packaging cells with pLNChGsn (Figure 1A

). An empty vector pLNCX containing only neomycin-resistance gene was generated as control virus (designated as RNV). The lung squamous cell carcinoma PC10 was infected with either RGV or RNV and selected with G418. Initially, six clones infected with RGV were designated as PG and four clones infected with RNV were designated as PN. They were randomly selected and gelsolin expression was confirmed against the parental cell clones on Western blot analysis. Gelsolin expressions in PG clones were higher than those of control cells, PN and PC10 (data not shown). PG2, PG3, PN3 and PN4 clones as well as PC10 were used for this experiment. Gelsolin expressions were verified on Western blot and quantified on densitometric analysis (Figure 1B, C). Gelsolin protein levels were about 5.0- and 7.5-fold higher in PG3 and PG2 cells, respectively, than the parental clone.

### Tumorigenicity of gelsolin-overexpressing clones *in vitro* and *in vivo*

**Figure 2:**
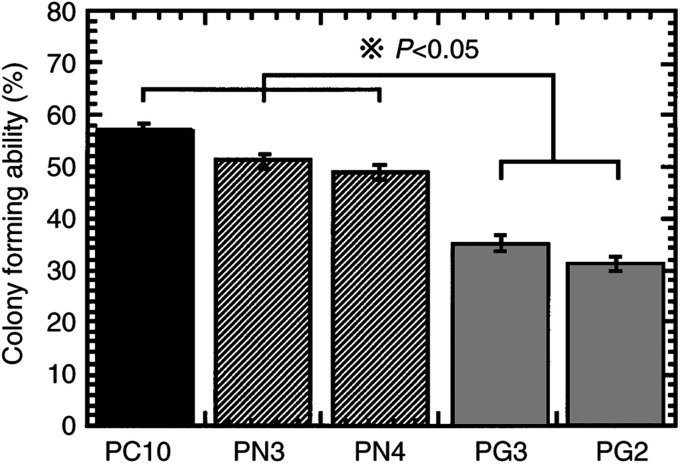
Effects of gelsolin expression on the colony-forming ability of transfectants in soft agar. Parental PC10, neo-transfectants PN3, PN4 and gelsolin transfectants PG3, PG2 cells were plated in 0.33% agar on 60 mm plates and the rate of colony forming at 3 weeks after incubation was analysed. ^*^P<0.05, compared with parental and neo-transfectant cells by *post hoc* test.

We next examined the effects of restoration of gelsolin expression on tumorigenicity of PC10 in soft agar and in nude mice. As shown in Figure 2

, colony formation was significantly reduced by overexpression of gelsolin in PC10 (*P*<0.05). Next, we tested the tumorigenecity of gelsolin-overexpressing clone PG2 and the neo-control clone PN3 in nude mice. The tumorigenicity of PG2 was suppressed as compared to PN3 in Exp. 1 (Table 1

**Table 1:** Tumorigenicity in nude mice of human lung cancer cell line (PC10) or infected with gelsolin expression virus (LNChGsn) or neo-control virus (LNCX)

	**Tumour incidence**		**Tumour volume at killing (days: 60, mean) (mm^3^)**	
**Cell line**	**Exp. 1**	**Exp. 2**	**Exp. 1**	**Exp. 2**
PC10		2/2		710
PN3	2/2	2/2	1128	1678
PG3		2/2		22
PG2	1/2	0/2^a^	242	0

**Figure 3:**
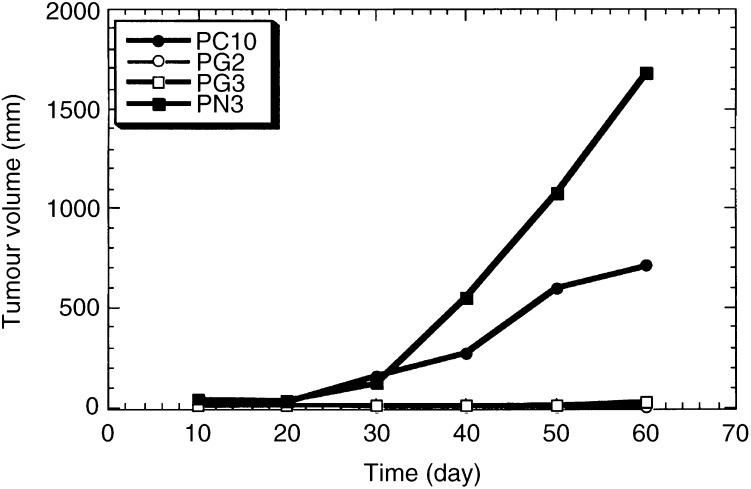
Effects of gelsolin expression on tumorigeneseity in nude mice. Cells (3×10^6^) were inoculated subcutaneously into the back of BALB/c-nu/nu syngenic 6-week-old male mice. Tumour size was measured using a hand calipers every 10 days as described in Materials and Methods.

The indicated cells were grown as described in Materials and Methods. Two mice per clone were injected subcutaneously into the back with 3×10^6^ cells in a final volume of 100 *μ*l of BS. Tumour size was measured using a hand calipers every 10 days. Volumes were calculated as described in Materials and Methods.

a Tumours were regressed at days 24 or 50 later Inoculation.

). In Exp. 2, the implanted PG2 and PG3 cells overexpressing gelsolin grew more slowly than PN3 control cells and PC10 parental cells in nude mice, indicating that tumorigenicity of PG2 and PG3 clones was strongly suppressed, in parallel with the amounts of expressed gelsolin (Figure 3

). Furthermore, PG2 cells regressed in both mice tested in Exp. 2 (Table 1). These results demonstrated that gelsolin functioned as a tumour suppressor in lung cancer cells, consistent with previous observations in bladder and colon cancers (Tanaka *et al*, 1995; Furuuchi *et al*, 1996).

### Cell growth and apoptosis *in vitro*

**Figure 4:**
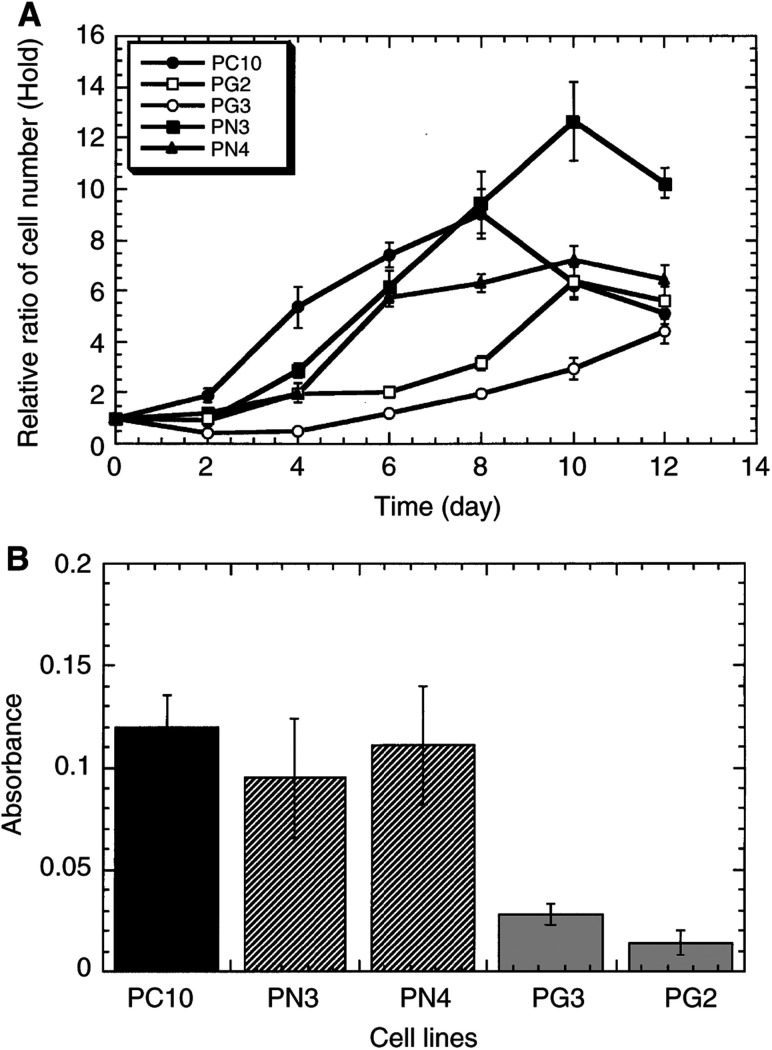
Growth properties of wild-type, neo- and gelsolin transfectant cell lines. (**A**) Growth curve of parent, neo- and gelsolin transfectants. Cells were seeded at a density of 5×10^4^ cells per well in six-well plates in RPMI 1640 with either 10 or 1% FCS as described in Materials and Methods. Cell number in triplicate wells was determined by counting with a haemocytometer after trypsinisation every 24 h. (**B**) Cell growth was also examined by MTT assay as described in Materials and Methods. Flat 96-well culture plates seeded at a density of 5×10^3^ cells per well were used to test growth with 1% FCS medium. The optical density of the plates was measured on a microculture plate reader using a test wavelength of 570 nm and a reference wavelength of 630 nm.

It has been proposed that the balance between cell proliferation and apoptosis determines tumour growth *in vivo* (Reed, 1999). We next studied *in vitro* whether gelsolin overexpression led to tumour regression because cell proliferation was restrained or because the apoptotic process was enhanced in PGs or both. The cell growths of the transfectants, control cells and parental cells were examined in a medium containing 10 or 1% FCS. The two clones PG2 and PG3 transfected with gelsolin cDNA grew more slowly than the control cells under 1% FCS condition (Figure 4A

). Under 10% FCS condition, there was no difference in the growth rates (data not shown). Similarly, MTT assay indicated that gelsolin transfection subdued cell growth (Figure 4B). In contrast, apoptotic rates assayed by counting cells sensitive to staurosporine were similar among the PGs, PNs and PC10 (data not shown). It was suggested that gelsolin suppressed tumour growth *in vivo* by affecting the cell-proliferating ability of PC10 rather than by inducing apoptosis.

### Inositol triphosphate (IP3) production in response to bradykinin treatment

**Figure 5:**
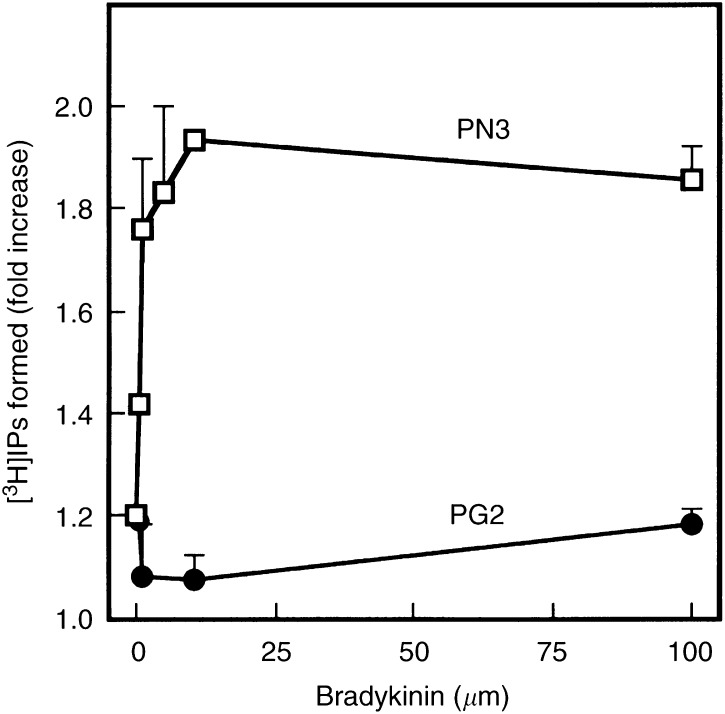
Effects of gelsolin overexpression on PLC activation induced by bradykinin. Vector (PN3)- or gelsolin (PG2)-overexpressing PG10 cells were labelled wtih [^3^H]inositol in inositol-free RPMI 1640 containing 0.3% BSA for 24 h. [^3^H]inositol phosphate generation was measured at indicated concentrations of bradykinin for 2 min as described in Materials and Methods. Results represent the mean±s.e. of three independent experiments.

Gelsolin exhibits an ability of binding to phosphatidylinositol 4,5-bisphosphate (PIP2), and inhibits PLC activity by competing with PIP2 (Banno *et al*, 1992; Sun *et al*, 1997). Moreover, gelsolin overexpression has recently been reported to suppress bradykinin-induced PLC and phospholipase D (PLD) in NIH3T3 cells (Banno *et al*, 1999). These results indicate that gelsolin functions as a modulator of lipid metabolism in fibroblast cells. As shown in Figure 5

, IP3 production by treatment with bradykinin was markedly inhibited in PG2, overexpressing gelsolin as compared to in the neo-control clone PN3, indicating that PLC activity was also suppressed by overexpression of gelsolin in the epithelial cell system.

### Translocation of PKC isoforms induced by bradykinin

**Figure 6:**
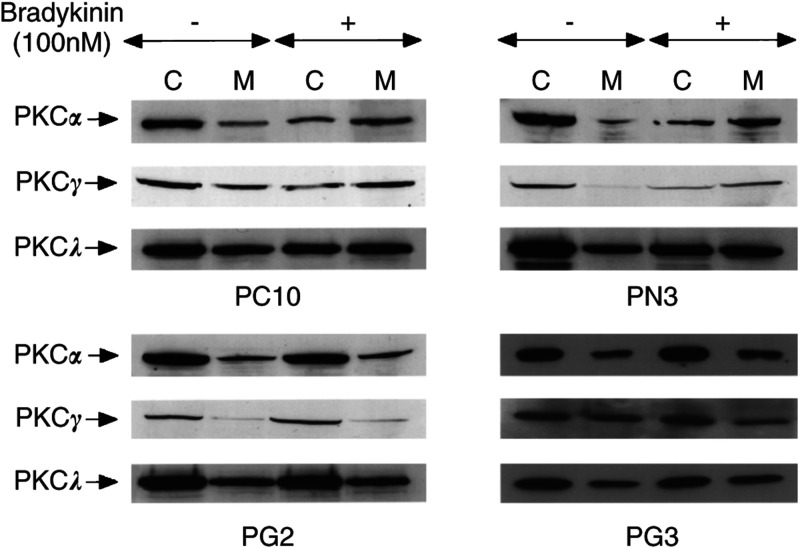
Bradykinin-induced PKCs translocation in transfectants by Western blot analysis. For activation studies, the bradykinin-induced translocation of PKC isoforms from the cytosolic fraction to the membrane fraction was investigated by cell fractionation as described in Materials and Methods. Cytosol and membrane fractions (20 *μ*g per lane) were separated with 10% polyacrylamide gel and analysed by Western blot analysis with monoclonal antibodies specific for PKC isoforms. The PKC proteins are represented by the arrows. C, cytosolic fractions; M, membrane fractions.

Among the different PKC isoforms, the conventional and the novel PKCs have been demonstrated to be activated by diacylglycerol (DAG) or phorbol esters or both (Toker, 1998). Activation of PKCs is a crucial step in the intracellular signal transduction pathway common to many growth factors (Hunter, 1997). Tumour cells with an increased PKC activity have an enhanced ability to invade and metastasise, indicating PKC roles critical to malignant phenotypes (Korczak *et al*, 1989; Schwartz *et al*, 1990). Western blotting of whole cell extracts showed that PC10 cells expressed the PKC isoforms *α*, *γ*, *ι* and *λ* (data not shown). Gelsolin inhibits the hydrolysis of PIP2 by PLC as described above, and thereby suppresses the generation of DAG. For studying the activation mechanism, we examined the stimulus-induced translocation of PKC isoforms from the cytosolic fraction to the particulate fraction. After treatment with 12-*O*-tetradecanoylphorbol-13-acetete (TPA) (1000 ng ml^−1^) for 1 h, the membrane fractions of conventional PKCs, PKC*α* and PKC*γ* increased in all cells, indicating no defect of the PKC pathway in transfectants overexpressing gelsolin as well as the neo-control clones and parental PC10 cells. In addition, the translocation of the atypical PKC isoforms (*ι* and *λ*) was not observed in all cells as expected (data not shown). As carried out in the other studies of bradykinin-induced translocation of PKC isoforms in different cell lines (Graness *et al*, 1998), PC10 and transfected cells were stimulated with 100 nM bradykinin for 5 min. The membrane fractions of PKC*α* and PKC*γ* increased in PC10 and PN3 cells when treated with bradykinin. On the other hand, in PG2 and PG3 cells treated with bradykinin, PKC*α* and PKC*γ* did not change in membrane fractions. Furthermore, the atypical PKC isoforms *λ* did not translocate in any clones when treated with bradykinin (Figure 6

). Our results suggested that gelsolin suppressed the activation of PKC by decreasing the production of DAG. Collectively, our results indicate that overexpression of gelsolin in PC10 cells causes tumour suppression in nude mice through inhibiting the activation of PKCs by sequestering PIP2, which is a substrate of PLC.

## DISCUSSION

In this study, we demonstrated that gelsolin suppressed tumorigenicity of PC10 lung cancer cells through inhibiting a PKC signal transduction pathway. Gelsolin is a representative of actin-regulatory proteins with an 82 kDa mass and is present in most vertebrate tissues (Kwiatkowski *et al*, 1988; Lueck *et al*, 1998). Its actions are regulated positively by calcium ions or protons, and negatively by polyphosphoinositides, especially by PIP2, suggested by the ability of gelsolin to bind to PIP2 (Janmey and Stossel, 1987; Janmey, 1994). We previously reported that gelsolin expression is reduced or not detected in various lung cancer cell lines and in more than half of the surgically resected tissues (Dosaka-Akita *et al*, 1998), and demonstrated that ectopic expression of gelsolin cDNA in bladder and colon cancer cell lines results in suppression of tumorigenicity (Tanaka *et al*, 1995; Furuuchi *et al*, 1996).

Activation of proto-oncogenes or inactivation of tumour suppressor genes by either mutation, amplification or rearrangement of DNA has been shown to be a critical step for carcinogenesis (Hunter, 1997). Tumour suppressor genes are classified into two categories, class I and class II. In class I tumour suppressor genes, usually isolated by positional cloning strategy, including RB, p53 or APC, loss of their function results from mutation or deletion of DNA. An increasing number of class II tumour suppressor genes have been identified on the basis of their downregulated expressions and of their transformation-inhibitory activity in tumour cells (Sager, 1997; Zhang *et al*, 1998). All these findings suggest that downregulation of gelsolin is involved in the development of various cancers as one of the early events in carcinogenesis, and that gelsolin may function as a tumour suppressor.

We established stable clones of lung cancer cells overexpressing various levels of gelsolin to investigate the effects of gelsolin restoration on tumorigenicity. In our studies, gelsolin-overexpressing lung cancer cells exhibited a significant growth inhibition under the low-level serum condition, consistent with our previous findings (Ishizaki *et al*, 1995), decreased the ability of colony formation in soft agar and reduced tumorigenicity in nude mice. PG2, highly expressing gelsolin, reduced and finally lost tumorigenicity, whereas PG3, moderately expressing gelsolin, slightly grew in nude mice. These findings demonstrated that gelsolin played a key role as a tumour suppressor in lung cancer cells and that the effect depended on the levels of gelsolin expression.

Agonist-stimulated activation of PLC *β*, *γ* and *δ* subtypes resulted in hydrolysis of membrane inositol phospholipid PIP2 (Williams, 1999), and led to the generation of DAG and soluble IP3. It is likely that gelsolin may affect the phospholipid signalling pathway after the hydrolysis of PIP2 supported by its high affinity for PIP2. PLC activity is also reported to be modulated in NIH3T3 cells overexpressing wild-type gelsolin or CapG, an actin-capping protein structurally related with gelsolin (Sun *et al*, 1995; Sun *et al*, 1997). Our group found that phospholipase D activity is affected by overexpression of human gelsolin cDNA in NIH3T3 cells via PLC/PKC pathway (Banno *et al*, 1999).

PKC isoforms appear to be localised in the cytoplasm before stimulation. For example, in MCF-7 cells, PKC*α* is present in the cytosol before stimulation, and 12-*O*-tetradecanoylphorbol-13-acetete (TPA) treatment induces translocation of PKC*α* from the soluble fraction to the particulate fraction (Kennedy *et al*, 1992). To demonstrate activation of PKCs in our experiments, their stimulus-induced translocation from the cytosol to the membrane was investigated. We identified two types of PKC isoforms in PC10 cells: conventional PKC (PKC*α* and PKC*γ*) and atypical PKC (PKC*ι* and PKC*λ*). By TPA treatment, translocation of cPKCs was observed in parental cells, control and gelsolin transfectants. However, aPKC translocation was not detected in any cells by the same stimulation.

**Figure 7:**
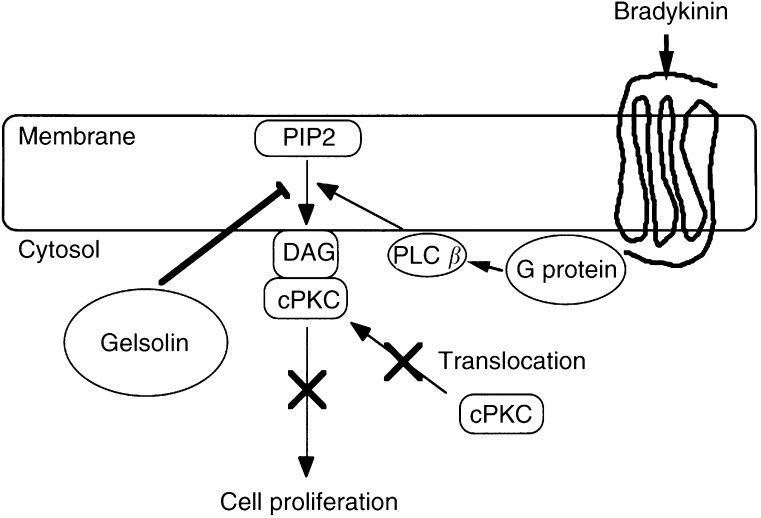
Model for the inhibition of bradykinin-induced cPKCs translocation by gelsolin in PC10 cells. Tumour promotion mostly depended on the PKC signal transduction pathway via the route that activated PLC*β* stimulated through a G-protein-coupled receptor. Gelsolin sequesters PIP2 and suppresses production of DAG and IP3 by PLC*β*, and thereby translocation to membrane fraction and activation of cPKCs is inhibited. As the PLC*β*/cPKCs pathway is critical for growth of lung cancer cells, and overexpression of gelsolin in PC10 cells results in the loss of tumorigenicty.

Bradykinin binds to a seven transmembrane domain receptor coupled with G-protein (Burch and Axelrod, 1987), activating PLC*β*. PLC*β* hydrolyses PIP2, resulting in the generation of DAG, which in turn activates PKC. As shown in Figure 4, translocation of PKC*α* and *γ*, but not that of PKC*ι*/*λ*, was observed in PC10 and PN3 in response to bradykinin treatment, whereas the translocation was hampered in PG2, suggesting that activation of PKC*α* and *γ* was inhibited in PG2. We also examined the translocation of PKCs in parental cells, control and gelsolin transfectants by epidermal growth factor (EGF) treatment. PIP2 is hydrolysed to DAG and IP3 by PLC*γ*, which EGF phosphorylates on its tyrosine residues (Goldschmidt-Clermont *et al*, 1991). However, we did not detect EGF-induced translocation of PKCs in either clone (data not shown). These results indicated that overexpression of gelsolin inhibited the PLC*β*/PKC pathway critical for the growth of PC10 cells, leading to the loss of tumorigenicity. Gelsolin could have a regulatory function in the lipid signalling pathway not only in the fibroblast system but also in epithelial cells. Tumour promotion in PC10 cells mostly depended on the PKC signal transduction pathway mediated by the activation of PLC*β*. Consistent with our findings, stable transfection of PKC*α* antisense was documented to decrease the malignant phenotypes of the parental cells (Wang *et al*, 1999). This observation supported our findings that gelsolin inhibited the activation of PKC in lung cancer cells and reduced their growth and tumorigenicity (Figure 7

).

In conclusion, we showed that, in PC10 cells, gelsolin suppressed the activation of PKCs involved in phospholipid signalling pathways and inhibited cell growth and tumorigenicity in nude mice. Further biological researches on gelsolin will certainly be required in order to exploit the tumour-suppressing effect of gelsolin expression in the clinical field. For the present, the p53 gene transfer using an adenovirus vector has been used as a tumour suppressor for gene therapy in nonsmall-cell lung cancers (Roth *et al*, 1999). The introduction of gelsolin into various cancers might possibly serve as an antitumour gene therapy in the future.

## References

[bib1] Banno Y, Fujita H, Ono Y, Nakashima S, Ito Y, Kuzumaki N, Nozawa Y. (1999) Differential phospholipase D activation by bradykinin and sphingosine 1-phosphate in NIH 3T3 fibroblasts overexpressing gelsolin. J Biol Chem 274: 27385–27391 10488069 10.1074/jbc.274.39.27385

[bib2] Banno Y, Nakashima T, Kumada T, Ebisawa K, Nonomura Y, Nozawa Y. (1992) Effects of gelsolin on human platelet cytosolic phosphoinositide-phospholipase C isozymes. J Biol Chem 267: 6488–6494 1313007

[bib3] Berridge MJ, Dawson RMC, Downes P, Heslop JP, Irvine RF (1983) Changes in the levels of inositol phosphates after agonist-dependent hydrolysis of membrane phosphoinositides. Biochem J 212: 473–482 6309146 10.1042/bj2120473 PMC1152070

[bib4] Borner C, Ueffing M, Jaken S, Parker PJ, Weinstein IB (1995) Two closely related isoforms of protein kinase C produce reciprocal effects on the growth of rat fibroblasts. Possible molecular mechanisms. J Biol Chem 270: 78–86 7814423 10.1074/jbc.270.1.78

[bib5] Burch RM, Axelrod J (1987) Dissociation of bradykinin-induced prostaglandin formation from phosphatidylinositol turnover in Swiss 3T3 fibroblasts: evidence for G protein regulation of phospholipase A2. Proc Natl Acad Sci USA 84: 6374–6378 2888113 10.1073/pnas.84.18.6374 PMC299078

[bib6] Cacace AM, Guadagno SN, Krauss RS, Fabbro D, Weinstein IB (1993) The epsilon isoform of protein kinase C is an oncogene when overexpressed in rat fibroblasts. Oncogene 8: 2095–2104 8336936

[bib7] Castagna M, Takai Y, Kaibuchi K, Sano K, Kikkawa U, Nishizuka Y (1982) Direct activation of calcium-activated, phospholipid-dependent protein kinase by tumor-promoting phorbol esters. J Biol Chem, 257: 7847–7851 7085651

[bib8] Dekker LV, Parker PJ (1994). Protein kinase C–a question of specificity. Trends Biochem Sci 19: 73–77 8160269 10.1016/0968-0004(94)90038-8

[bib10] Dosaka-Akita H, Hommura F, Fujita H, Kinoshita I, Nishi M, Morikawa T, Katoh H, Kawakami Y, Kuzumaki N (1998) Frequent loss of gelsolin expression in non-small cell lung cancers of heavy smokers. Cancer Res 58: 322–327 9443412

[bib11] Furuuchi K, Fujita H, Tanaka M, Shinohara N, Senmaru N, Ogiso Y, Moriya S, Hamada M, Kato H, Kuzumaki N (1996) Gelsolin as a suppressor of malignant phenotype in human colon cancer. Tumor Target 2: 277–283

[bib12] Goldschmidt-Clermont PJ, Kim JW, Machesky LM, Rhee SG, Pollard TD (1991) Regulation of phospholipase C-*γ*1 by profilin and tyrosine phosphorylation. Science 251: 1231–1233 1848725 10.1126/science.1848725

[bib13] Gopalakrishna R, Barsky SH (1988) Tumor promoter-induced membrane-bound protein kinase C regulates hematogenous metastasis. Proc Natl Acad Sci USA 85: 612–616 3422445 10.1073/pnas.85.2.612 PMC279601

[bib14] Graness A, Adomeit A, Heinze R, Wetzker R, Liebmann C (1998) A novel mitogenic signaling pathway of bradykinin in the human colon carcinoma cell line SW-480 involves sequential activation of a Gq/11 protein, phosphatidylinositol 3-kinase beta, and protein kinase C epsilon. J Biol Chem 273: 32016–32022 9822674 10.1074/jbc.273.48.32016

[bib15] Housey GM, Johnson MD, Hsiao WL, O'Brian CA, Murphy JP, Kirschmeier P, Weinstein IB (1988) Overproduction of protein kinase C causes disordered growth control in rat fibroblasts. Cell 52: 343–354 3345563 10.1016/s0092-8674(88)80027-8

[bib16] Hunter T (1997) Oncoprotein networks. Cell 88: 333–346 9039260 10.1016/s0092-8674(00)81872-3

[bib17] Ishizaki A, Fujita H, Kuzumaki N (1995) Growth-inhibitory functions of a mutated gelsolin (His321) in NIH/3T3 mouse fibroblasts. Exp Cell Res 217: 448–452 7698245 10.1006/excr.1995.1108

[bib18] Janik P, Szaniawska B, Kowalczyk D, Miloszewska J (1994) The effect of phorbol ester treatment on migration of C3H 10T1/2 and BT5C glioma cells: possible application to carcinogenesis. J Cancer Res Clin Oncol 120: 156–158 8263011 10.1007/BF01202194 PMC12200044

[bib19] Janmey PA (1994) Phosphoinositides and calcium as regulators of cellular actin assembly and disassembly. Annu Rev Physiol 56: 169–191 8010739 10.1146/annurev.ph.56.030194.001125

[bib20] Janmey PA, Stossel TP (1987) Modulation of gelsolin function by phosphatidylinositol 4,5-bisphosphate. Nature 325: 362–364 3027569 10.1038/325362a0

[bib21] Johnson MD, Torri JA, Lippman ME, Dickson RB (1999) Regulation of motility and protease expression in PKC-mediated induction of MCF-7 breast cancer cell invasiveness. Exp Cell Res 247: 105–113 10047452 10.1006/excr.1998.4336

[bib22] Kennedy MJ, Prestigiacomo LJ, Tyler G, May WS, Davidson NE (1992) Differential effects of bryostatin 1 and phorbol ester on human breast cancer cell lines. Cancer Res 52: 1278–1283 1737390

[bib23] Kishimoto A, Takai Y, Mori T, Kikkawa U, Nishizuka Y (1980) Activation of calcium and phospholipid-dependent protein kinase by diacylglycerol, its possible relation to phosphatidylinositol turnover. J Biol Chem 255: 2273–2276 7358670

[bib24] Korczak B, Whale C, Kerbel RS (1989) Inhibition of invasion of invasive human bladder carcinoma cells by protein kinase C inhibitor staurosporine. Cancer Res 49: 2597–2602 2496916

[bib25] Kwiatkowski DJ, Mehl R, Izumo S, Nadal GB, Yin HL (1988) Muscle is the major source of plasma gelsolin. J Biol Chem 263: 8239–8243 2836420

[bib26] Lueck A, Brown D, Kwiatkowski DJ (1998) The actin-binding proteins adseverin and gelsolin are both highly expressed but differentially localized in kidney and intestine. J Cell Sci 111: 3633–3643 9819354 10.1242/jcs.111.24.3633

[bib27] Mellor H, Parker PJ (1998) The extended protein kinase C superfamily. Biochem J 332: 281–292 9601053 10.1042/bj3320281 PMC1219479

[bib28] Mischak H, Goodnight JA, Kolch W, Martiny-Baron G, Schaechtle C, Kazanietz MG, Blumberg PM, Pierce JH, Mushinski JF (1993) Overexpression of protein kinase C-delta and -epsilon in NIH 3T3 cells induces opposite effects on growth, morphology, anchorage dependence, and tumorigenicity. J Biol Chem 268: 6090–6096 8454583

[bib29] Moriya S, Yanagihara K, Fujita H, Kuzumaki N (1994) Differential expression of hsp90, gelsolin and GST-*π* in human gastric carcinoma cell lines. Int J Oncol 5: 1347–1351 21559720 10.3892/ijo.5.6.1347

[bib30] Nishizuka Y (1984) The role of protein kinase C in cell surface signal transduction and tumour promotion. Nature 308: 693–698 6232463 10.1038/308693a0

[bib31] Nishizuka Y (1992) Intracellular signaling by hydrolysis of phospholipids and activation of protein kinase C. Science 258: 607–614 1411571 10.1126/science.1411571

[bib32] Otterson GA, Xiao GH, Geradts J, Jin F, Chen WD, Niklinska W, Kaye FJ, Yeung RS (1998) Protein expression and functional analysis of the FHIT gene in human tumor cells. J Natl Cancer Inst 90: 426–432 9521166 10.1093/jnci/90.6.426

[bib33] Persons DA, Wilkison WO, Bell RM, Finn OJ (1988) Altered growth regulation and enhanced tumorigenicity of NIH 3T3 fibroblasts transfected with protein kinase C-I cDNA. Cell 52: 447–458 3162207 10.1016/s0092-8674(88)80037-0

[bib34] Reed JC (1999) Mechanisms of apoptosis avoidance in cancer. Curr Opin Oncol 11: 68–75 9914881 10.1097/00001622-199901000-00014

[bib35] Rotenberg SA, Sun XG (1998) Photoinduced inactivation of protein kinase C by dequalinium identifies the RACK-1-binding domain as a recognition site. J Biol Chem 273: 2390–2395 9442087 10.1074/jbc.273.4.2390

[bib36] Roth JA, Swisher SG, Meyn RE (1999) p53 tumor suppressor gene therapy for cancer. Oncology (Huntingt) 13: 148–154 10550840

[bib37] Sager R (1997) Expression genetics in cancer: shifting the focus from DNA to RNA. Proc Natl Acad Sci, USA 94: 952–955 9023363 10.1073/pnas.94.3.952 PMC19620

[bib38] Salgia R, Skarin AT (1998) Molecular abnormalities in lung cancer. J Clin Oncol 16: 1207–1217 9508209 10.1200/JCO.1998.16.3.1207

[bib39] Schwartz GK, Redwood SM, Ohnuma T, Holland JF, Droller MJ, Liu BC (1990) Possible involvement of Ca^2+^ mobilization and protein kinase C activation in the induction of spontaneous metastasis by mouse mammary adenocarcinoma cells. J Natl Cancer Inst 82: 1753–17562231770

[bib40] Sun H, Lin K, Yin HL (1997) Gelsolin modulates phospholipase C activity *in vivo* through phospholipid binding. J Cell Biol 138: 811–820 9265648 10.1083/jcb.138.4.811 PMC2138049

[bib41] Sun HQ, Kwiatkowska K, Wooten DC, Yin HL (1995) Effects of CapG overexpression on agonist-induced motility and second messenger generation. J Cell Biol 129: 147–156 7698981 10.1083/jcb.129.1.147 PMC2120377

[bib42] Tanaka M, Müllauer L, Ogiso Y, Fujita H, Moriya S, Furuuchi K, Harabayashi T, Shinohara N, Koyanagi T, Kuzumaki N (1995) Gelsolin: a candidate for suppressor of human bladder cancer. Cancer Res 55: 3228–3232 7614452

[bib43] Toker A (1998) Signaling through protein kinase C. Front Biosci 3: D1134–1147 9792904 10.2741/a350

[bib44] Toker A, Meyer M, Reddy KK, Falck JR, Aneja R, Aneja S, Parra A, Burns DJ, Ballas LM, Cantley LC (1994) Activation of protein kinase C family members by the novel polyphosphoinositides PtdIns-3,4-P2 and PtdIns-3,4,5-P3. J Biol Chem 269: 32358–32367 7798235

[bib45] Wang XY, Repasky E, Liu HT (1999) Antisense inhibition of protein kinase C-alpha reverses the transformed phenotype in human lung carcinoma cells. Exp Cell Res 250: 253–263 10388539 10.1006/excr.1999.4529

[bib46] Williams RL (1999) Mammalian phosphoinositide-specific phospholipase C. Biochim Biophys Acta 1441: 255–267 10570253 10.1016/s1388-1981(99)00150-x

[bib47] Workman P, Twentyman P, Balkwill F, Balmain A, Chaplin D, Double J, Embleton J, Newell D, Raymond R, Stables J, Stephens T, Wallace J (1998) United Kingdom Co-ordinating Committee on Cancer Research (UKCCCR) guidelines for the welfare of animals in experimental neoplasia. Br J Cancer 77: 1–10 10.1038/bjc.1998.1 PMC2151254 9459138

[bib48] Yin HL, Stossel TP (1979) Control of cytoplasmic actin gel-sol transformation by gelsolin, a calcium-dependent regulatory protein. Nature 281: 583–586 492320 10.1038/281583a0

[bib49] Zhang M, Martin KJ, Sheng S, Sager R (1998) Expression genetics: a different approach to cancer diagnosis and prognosis. Trends Biotech 16: 66–71 10.1016/s0167-7799(97)01157-8 9487733

